# Mitonuclear Interactions in the Maintenance of Mitochondrial Integrity

**DOI:** 10.3390/life10090173

**Published:** 2020-08-31

**Authors:** Panagiotis Karakaidos, Theodoros Rampias

**Affiliations:** Biomedical Research Foundation of the Academy of Athens, 11527 Athens, Greece; pkarak@bioacademy.gr

**Keywords:** translational fidelity, mitochondrion genetic code, mt-DNA repair, mitonuclear coevolution, mitochondrial diseases

## Abstract

In eukaryotic cells, mitochondria originated in an α-proteobacterial endosymbiont. Although these organelles harbor their own genome, the large majority of genes, originally encoded in the endosymbiont, were either lost or transferred to the nucleus. As a consequence, mitochondria have become semi-autonomous and most of their processes require the import of nuclear-encoded components to be functional. Therefore, the mitochondrial-specific translation has evolved to be coordinated by mitonuclear interactions to respond to the energetic demands of the cell, acquiring unique and mosaic features. However, mitochondrial-DNA-encoded genes are essential for the assembly of the respiratory chain complexes. Impaired mitochondrial function due to oxidative damage and mutations has been associated with numerous human pathologies, the aging process, and cancer. In this review, we highlight the unique features of mitochondrial protein synthesis and provide a comprehensive insight into the mitonuclear crosstalk and its co-evolution, as well as the vulnerabilities of the animal mitochondrial genome.

## 1. Eukariotic Cell: A Chimeric Union with Two Genomes

The endosymbiont hypothesis is widely accepted for its ability to explain the mitochondrial origin [1,2]. Complete sequencing of the mitochondrial genome (mtDNA) from several mammalian species revealed that mtDNA is of α-proteobacterial origin and that all extant mtDNAs have originated from the same ancestral bacterial genome [3,4]. According to these early sequencing studies, mtDNA encodes a small number (13 in mammals) of protein subunits of the mitochondrial electron transport chain and ATP synthase. It also encodes the ribosomal RNA (rRNA) and transfer RNA (tRNA) components of the mitochondrial translation system. 

Two basic endosymbiotic models, namely, the “archezoan scenario” and the “symbiogenesis scenario,” have been proposed [5]. According to the archezoan scenario, the mitochondrion evolved from an α-proteobacterial ancestor via symbiosis within a primitive eukaryotic host cell termed an “archezoan.” Although controversy persists regarding the nature of the proto-eukaryotic host that engulfed the mitochondrion, members of the archezoa were probably primitive protists living as parasites in anaerobic environments and characterized by the absence of recognizable mitochondria [6]. 

An alternative view claiming that the host cell for the mitochondrial endosymbiosis was an archaebacterion is the basis of the “symbiogenesis scenario.” According to this model, the evolution of the nucleus and the compartmentalization of the eukaryotic cell happened after a single endosymbiotic event [7,8]. Phylogenomic analyses that support the symbiogenesis scenario provide evidence that the host cell for endosymbiosis is closely related to a group of Archaea known as the Asgards [9,10].

The transition from endosymbiotic bacterium to a semi-autonomous permanent organelle included many major evolutionary events, such as genome reduction, the origin of a protein-importing system, and the insertion of membrane transporters. Whether the α-proteobacterium provided a metabolic advantage to the host cell, at the early steps of endosymbiosis, has been a matter of controversy [11,12]. However, it is widely accepted that mitochondria were established and retained as organelles because of their capacity to efficiently generate ATP through aerobic respiration.

The mechanisms and evolutionary forces that shaped mitochondrial organellogenesis were diverse among the branches of the eukaryote tree, and for this reason, a high level of diversity is observed between mitochondrial genomes and proteomes in eukaryotes [13]. For instance, many distinct eukaryotic lineages have adapted to living in low oxygen conditions, and as a result, many of these organisms have evolved mitochondria to function anaerobically [14].

## 2. Mitochondrial Genome: Evolving While Keeping Essentials

Mitochondria consist of approximately 1400 different proteins, which are located in the outer membrane, inner membrane, intermembrane space, and matrix. However, mitochondrial genomes are severely degenerated, encoding only a few proteins, while the majority of mitochondrial proteins are encoded from genes that have been transferred to the host nuclear genome during endosymbiosis [15]. However, this limited genetic information of mtDNA is well conserved across eukaryotes. In humans, the mitochondrial genome codes for only 13 core subunits of the respiratory chain, while the rest of the approximately one thousand mitochondrial proteins are encoded by nuclear genes and translated on cytosolic ribosomes with targeting signals before being imported into mitochondria [16]. At least five different protein-importing pathways have been identified so far, based on translocase complexes that recognize different targeting signals to mediate protein import into mitochondrial membranes [17,18].

A similar functional gene transfer into the nucleus has also been observed in chloroplasts [19]. Gene transfer from organelles to the nucleus is possibly a general feature of the endosymbiosis process that favors the assembly of all available genetic information in the nucleus [20]. This evolutionary tension to reduce the mitochondrial genome includes the loss of nonessential sequences and the transfer of essential genes from the mitochondrial genome to the nuclear one. The driving force for this transfer has been traditionally linked to the high mutation rate of mitochondrial genomes, which creates strong selective pressure for the transfer of essential genes to the nuclear genome, where they are protected by sexual reproduction and recombination [21,22]. Other proposed selective advantages of the nuclear encoding of organelle genes include the protection from mitochondrial mutagens [23] and the replication advantage of shorter mitogenomes. Given that mtDNA replicates independently of the cell cycle, the existence of shorter mtDNA molecules provides a replication advantage. In this direction, it has also been proposed that gene transfer to the nucleus may also represent a strategy employed by mtDNA to increase its replication potential by shortening its replication time [24].

The reason why mitochondria retain a small-sized genome that encodes a low number of proteins, despite the high rate of gene transfer to the nucleus, remains controversial. The mitochondrial genome uses a genetic code in which few of the 64 possible codons have a different meaning than in the nuclear genome. The hypothesis that the transfer is not permissive for regions that cannot be correctly decoded when translated to the cytosol is probably the older one [25,26]; however, this explanation does not apply to all mitochondrial genomes. Moreover, this proposed model suffers from the observation that a plethora of mitochondrial sequences with predicted decoding problems by the standard code has already been transferred to the nucleus. Another hypothesis for the retention of mitochondrial genes is that the extreme hydrophobicity of the mitochondria-encoded proteins could prevent their efficient import into the mitochondrion and would probably drive their relocation to the endoplasmic reticulum (ER). According to this hypothesis, the assembly of oxidative phosphorylation (OXPHOS) complexes in the mitochondrial inner membrane can be efficiently performed only when the OXPHOS polypeptides are co-expressed in the mitochondrion [27,28]. In support of this hypothesis, experimental studies in yeast have demonstrated that cytochrome *b* polypeptide fused to the N-terminal mitochondrial targeting sequence of the ATPase subunit 9 is unable to be imported into yeast mitochondria [29]. Recently, Bjorkholm and colleagues investigated the subcellular localization pattern of the 13 human mtDNA-encoded proteins when expressed in the cytoplasm of HeLa cells. These experiments showed that 12 of the 13 proteins were localized to the ER, and only one protein, ATP8, was found in the mitochondrion, despite the inclusion of the same mitochondrial targeting sequence in all 13 proteins [30]. However, the hydrophobicity hypothesis has also been challenged on the basis that several nuclear-coded hydrophobic proteins have been identified in mitochondria, indicating an efficient import across the mitochondrial membrane, despite their high hydrophobicity [31]. The possible toxicity of some mitochondrial gene products in the cytosol has also been proposed to explain the evolutionary persistence of a mitochondrial genome [32].

The hypothesis that the encoded proteins in respiratory electron transport may sense the local redox state and regulate their gene expression accordingly in response to metabolic demands is currently popular and is termed the CORR hypothesis (co-location of genes and gene products for redox regulation of gene expression) [33,34,35].

The mitogenome of most metazoans is normally a single circular molecule that is 12–18 kb in size with a very compact organization of genetic information. In mammals, the mitogenome contains 13 genes that encode essential polypeptides of the OXPHOS system and 24 RNA genes (2 ribosomal RNAs and 22 tRNAs) that are required for mitochondrial protein synthesis. There is a lack of introns, while the coding sequences can be separated only by a few non-coding bases [3]. Furthermore, in some cases, there is an overlap in mitochondrial coding sequences for proteins (MT-ATP8/6 and MT-ND4/4L) or mitochondrial tRNA genes (mt-tRNA^Tyr^/mt-tRNA^Cys^) [36]. 

Unlike the nuclear genome, mitochondrial DNA exists in 1000–10,000 copies per cell, allowing for the accumulation of mutations. It is widely accepted that mt-DNA evolves at a much faster rate compared to the nuclear genome. The average ratio of the mitochondrial mutation rate over the nuclear mutation rate is above 20 in vertebrates [37]. Different biological processes may contribute to this ratio. As mitochondria have a central bioenergetic role in eukaryotic cells, their genome is expected to be exposed to excessive levels of mutagenic products of aerobic respiration, such as reactive oxygen species (ROS) [38]. However, whether the increased exposure to ROS is the leading mutagenic force on mtDNA has not been clearly demonstrated (discussed in Section 5.2) 

Due to mutation acquisition, the mitogenome population constantly varies (heteroplasmy) and is subjected to genetic selection, as some mitogenomes may segregate more frequently than others [39,40]. For instance, mitogenomes that replicate more often are transmitted more efficiently. Moreover, positive selection can influence the inheritance, favoring mitogenomes that provide more energy. On the other hand, since mitochondrial functions are essential for cellular metabolism and variation in the mitochondrial protein-coding sequence can directly influence the metabolic performance, selection pressure has been proposed to act on the removal of mitogenomes with deleterious mutations (discussed in Section 6). Of note, only maternally derived mitogenomes are transmitted to the animal offsprings and this pattern of inheritance is known as “maternal inheritance” [41,42].

## 3. Mitonuclear Coordination of Mitochondrial Translation

The reduction of the mitochondrial genome via gene transfer to the nucleus has led to a partial loss of essential components of the translation machinery. Once translated into the cytosol, these missing mitochondrial components must be imported to the mitochondrion to assemble a translation apparatus, which are adapted to execute protein synthesis on a small set of mitochondrial genes. Mitochondrial proteins are assembled at their ribosomes (mitoribosomes). The translation process is highly similar to cytosolic translation. The major difference in the two processes is the structure of the mitoribosome, which consists of a large and a small cytosolic subunit, but has a distinct size and a higher protein-to-rRNA ratio [43]. This is the result of the shrinkage of the mitogenome during evolution. The human mitoribosome (55S) is composed of a 28S small subunit and a 39S large subunit [44].

In this context, it is typical for the mitogenomes to have lost several tRNA-coding genes. The high variability of tRNA loss and the fact that it is not consistent with the assigned phylogenetic positions probably indicates that this occurred during multiple independent events and was irreversible. This loss is compensated by the import of several nucleus-encoded tRNA molecules [45]. However, the specificity to which individual tRNAs are imported differs greatly between organisms and might reflect the fundamental differences in the set of tRNAs that have remained on respective mitogenomes and the mechanisms underlying tRNA importing. As part of the evolutionary process, the import of cytosolic tRNAs must have preceded the loss of mitochondrial tRNA genes. 

The complete sequencing of mitogenomes from different species indicates that a variable, minimal set of tRNA genes, restricted to 20–22 species, is encoded by a mitogenome in almost all metazoans [46]. 

Based on comparisons between the DNA sequences of several mitochondrial protein genes and the actual amino acid sequences of the corresponding proteins, early studies indicated that the mammalian mitochondrial genetic code displays variations from the “universal” code. The reassignment of UGA from a termination codon to one coding Trp is a striking example [47]. Other variations include the non-universal codons AUA for Met and AGR (R = A or G) for the stop (termination) codon. Mammalian mitochondrial genetic code consists of 60 sense codons and the analysis of base-pairing rules between the anticodon and codon suggests that the full set of sense codons are deciphered mainly by the set of 22 mitochondrial tRNAs. In this context, this minimalistic set of tRNAs decodes an increased number of codons. For instance, tRNA^Met^, which in the canonical genetic code, recognizes only AUG codons; in mammalian mitochondria, it also binds to AUA codons, and in the case of the NADH dehydrogenase subunit 2 (*ND2*), it also binds to AUU.

Generally, the wobble rule is greatly simplified in the mitochondrial genetic code. Position 34, or the wobble base, in mt-RNAs is often occupied by an unmodified uridine that is capable of base pairing with any of the four bases due to the enhanced conformational flexibility within the anticodon loop [48]. As a consequence, each mt-tRNA decodes two to four codons in a family four-codon box, leading to an analogy of a single mitochondrion-encoded tRNA for every amino acid, except for leucine and serine, which are decoded by two tRNAs. 

Notably, mt-tRNAs constitute the smallest set of tRNAs with a complete decoding capacity among all kingdoms of life. This set has been adapted to decode approximately 4000 codons that correspond to the total length of the protein-encoded mitochondrial genes. Furthermore, decoding in mitochondria has been adapted for the translation of proteins with specific features. For instance, the AUA codon reassignment from isoleucine to methionine observed in most mitochondrial lineages leads to an increased methionine content in mitochondrial proteins that counteract the oxidative conditions at the inner membrane. This adaptation may also have provided advantages to eukaryotic host cells as it has been proposed that methionine is an evolutionarily selected antioxidant that protects respiratory chain complexes [49].

Recent yeast and human experimental data have demonstrated that even the mitochondria of these eukaryotes, which encode a presumably complete set of tRNAs, can also import cytosolic tRNAs. In *Saccharomyces cerevisiae*, after the early observation of the presence of a cytoplasmic tRNA^Lys^ (CUU) in mitochondria, both the cytosolic glutaminyl-tRNA synthetase (GlnRS) and the nucleus-encoded cytosolic tRNA^Gln^ were also found to be localized in mitochondria [50]. Moreover, imported cytosolic GlnRS was able to attach glutamine to cytosolic tRNA^Gln^ for mitochondrial protein synthesis. Similarly, nucleus-encoded tRNA^Gln^ (CUG) was found to be imported into rat and human mitochondria [51,52].

Bacterial and cytosolic eukaryotic tRNAs fold into a cloverleaf secondary structure, while their three-dimensional L-shape structure is stabilized by tertiary interactions to fit onto the ribosome’s A- and P-sites. Comparative analysis of the 22 mt tRNA genes from 31 mammals allowed for identifying particular structural features of these tRNAs. Most animal mitochondrial tRNAs have unusual secondary structures. Deviations from the canonical cloverleaf includes elongated anticodon stems, as well as changes in the number of nucleotides at the connectors and shortening of the D- and T-stems and loops. Moreover, helical regions display mismatches and tertiary base pairs are lost [53,54]. Among mammalian mitochondrial tRNA families, serine isoacceptor tRNAs from mammalian mitochondria have the most unusual secondary structures. More specifically, mitochondrial tRNA^Ser^ (GCU) probably has the structure that deviates the most, as it is missing the entire D-arm [55,56], while the mitochondrial tRNA^Ser^ (UGA) displays a cloverleaf structure with an elongation to the anticodon stem by one base pair, which leads to severe deformations of the tertiary interactions in the D-stem [57]. The length variation in D-stem needs to be compensated for by corresponding changes in other domains. Indeed, molecular modeling studies have demonstrated that the L-shaped tertiary structure in mitochondrial tRNAs with an anticodon stem elongation is preserved by shortening the connector regions or by multiple base deletions in the D and extra loop regions [57,58].

Interactions of the D- and T-loops in the standard tRNA structure have a critical role in the stabilization of the juxtaposition of the two helical domains. While a typical D-loop size in classical tRNAs is 8–10 nucleotides (nt) [59], the average and most frequently detected D-loop size in mammalian mt-tRNAs is 5 nt. Similarly, while a 7 bp T-loop size in canonical tRNAs is well conserved, T-loops as small as 1–2 nt and as large as 10 nt have been detected in mammalian mitochondrial tRNAs [53].

Moreover, alterations on conserved nucleotides (e.g., G18, G19, and T54) that are involved in tertiary interactions between D- and T-loops lead to large variations in D- and T-loop sizes. Additionally, all of the mammalian mitochondrial tRNAs have a short variable loop [53]. Furthermore, the nucleotide bias present in the mitochondrial genome leads to transcription of mt-tRNAs that are A-, U-, and C-rich, while G-poor, and are therefore stabilized by fewer stem G-C pairs compared to canonical tRNAs. As a consequence, the thermodynamic stability of mitochondrial tRNAs is predicted to be half as much (melting temperatures in the range of 50–60 °C) as those of canonical cytosolic tRNAs [60,61].

Post-transcriptional modifications by nuclear-encoded tRNA-modifying enzymes are likely to be of greater importance in mitochondrial tRNAs, given they have a specific role in their proper folding to a cloverleaf formation and in their aminoacylation [62]. For instance, a single methylation reaction of the adenosine residue at position 9 (m1A9) triggers the stabilization of the cloverleaf structure by blocking the base pairing between A9 and U64 in the T-stem. While this modification is highly abundant in mitochondrial RNAs, it is almost absent on cytosolic tRNAs.

These mitochondrial RNA modifications may include the addition of a simple group (e.g., methyl) or more complex chemical changes, such as a substitution (e.g., uridine to 4-thiouridine), oxidation/reduction (e.g., 5-methylcytidine to 5-hydroxymethylcytidine, uridine to dihydrouridine) and isomerization (e.g., uridine to pseudouridine). 

In contrast to the first and second codon–anticodon base pair, the ribosome imposes fewer restraints on the wobble base pair, and therefore various wobble pair structures; modifications can also be fitted in the decoding center [63]. In this context, numerous studies have demonstrated that wobble modifications at the tRNA anticodon loop can modulate codon recognition by restricting, expanding, or altering the decoding properties of the tRNAs [64].

**Figure 1:**
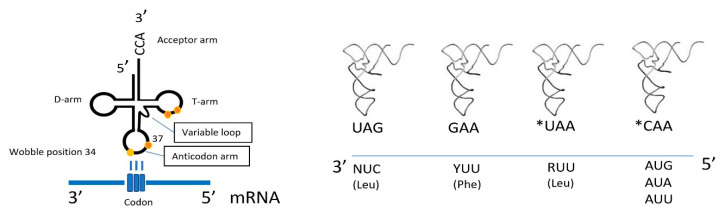
Schematic representation of codon–anticodon pairing and decoding in the mitochondrial translation process. Nucleotide modifications on the tRNA structure are indicated as color circles. *U and *C represent chemically modified forms of uridine and cytosine at the wobble position.

Interestingly, modifications at the anticodon loop of mitochondrial RNAs can also support the codon reassignments in mitochondria. As mentioned above, the single tRNA^Met^ in mammalian mitochondria recognizes both the AUA and AUG codons as Met, even though the anticodon CAU of tRNA^Met^ cannot base pair with the AUA codon, according to the conventional Watson–Crick pairing [65]. Moriya and colleagues identified a novel modified nucleoside, 5-formylcytidine (f5C), in the first position of the anticodon of mitochondrial tRNA^Met^ from a bovine liver that can pair with not only G but also with A in the third position of the codon [66]. Furthermore, chemically modified forms of uridine at the wobble position have recently been identified in mt-tRNA^Leu^ (5-taurinomethyluridine) and mitochondrial tRNA^Lys^ (5-taurinomethyl-2-thiouridine), which enhance the decoding fidelity of purine-ending NNG codon sets. Similarly, methylguanosine (m1G37) is frequently present at position 37 in mammalian mitochondrial tRNAs and has a critical role for tRNAs reading CNN codons [67]. A schematic representation of the special features of mt-DNA decoding is shown in Figure 1. 

The accuracy of protein synthesis is provided by aminoacyl-tRNA synthetases (ARSs) that direct tRNA charging via correct amino acids. This process critically depends on the ARSs’ ability to strictly discriminate cognate from non-cognate tRNAs. Two major factors seem to play a crucial role in ARS:tRNA recognition: (1) the tRNA fold and (2) a small fraction of nucleotides, which are termed identity and anti-identity elements [68,69]. In the case of mischarging, some synthetases also harbor an editing activity for hydrolyzing the mischarged amino acid [70].

During the evolution of the ancestral mitochondrial genome, all genes encoding ARSs were lost or transferred into the nucleus. As a consequence, all ARSs that function in the mitochondrial protein synthesis (mtARSs) are nucleus-encoded and post-translationally imported into the organelle.

**Figure 2:**
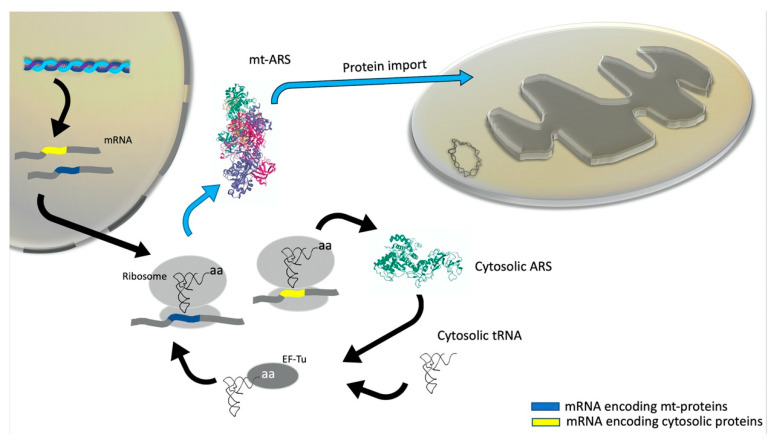
Features of human mtARS synthesis and translocation, where mtARSs are aminoacyl-tRNA synthetases (ARSs) that function in the mitochondrial protein synthesis. aa: Amino acid, EF-Tu: Elongation Factor Tu.

In humans, there are two sets of distinct nuclear genes: one set corresponding to cytosolic ARSs the other one to mitochondrial ARSs [71]. The only two exceptions are for GlyRSs and LysRSs, for which cytosolic and mitochondrial forms are generated either from two translation initiation sites or by alternative mRNA splicing of the same genes, respectively [72,73]. Human mt-aaRSs are translated within the cytosol and the mitochondrial targeting sequence (MTS), located at the N-terminus of the enzymes, drives their mitochondrial import [71] (Figure 2).

**Figure 3:**
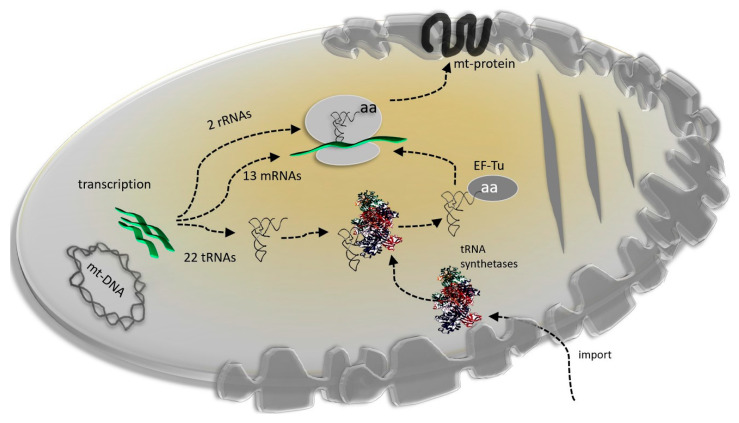
Schematic representation of the major components of mitochondrial translation.

As mentioned above, mt-tRNAs are characterized by an altered structure compared to cytosolic tRNAs, which includes weak base pairs, mismatches in the stem regions, reduced loop sizes, and reduced stability combined with the loss of canonical tRNA identity elements for aminoacylation [74]. Since all the nucleotide modification enzymes and ARSs are imported from the cytoplasm, codon reassignment in metazoan mitochondria would have been caused by the co-evolution of tRNA derived from the mitogenome and proteins derived from the nuclear genome (Figure 3).

Mitoribosomes are responsible for protein synthesis in mitochondria. Despite the proteobacterial origin of mitochondria, mitoribosomes are diverse in terms of their protein and rRNA content in different species [75,76]. The mammalian 55S mitoribosome consists of the small 28S subunit (mt-SSU) that binds the mitochondrial mRNAs and the large 39S subunit (mt-LSU) that catalyzes the peptide bond formation. Due to the endosymbiotic origin of mitochondria, it was expected that the mitoribosome would display a higher structural similarity to the bacterial ribosome than to the eukaryotic cytoplasmic ribosome. However, cryo-electron microscopy (cryo-EM) and biochemical studies have revealed a divergent structure and ribonucleoprotein composition compared to the bacterial ribosome [77,78,79]. The protein-to-RNA ratio is completely reversed in the animal mitoribosome (69% protein and 31% RNA) compared to bacterial ribosomes (33% protein and 67% RNA). As a result, the animal mitoribosome is characterized by reduced secondary RNA structures and longer protein–protein bridges. For instance, the 23S rRNA helix 38, which in the bacterial ribosome forms one of the RNA–protein intersubunit bridges that make important contacts with both A- and P-site tRNAs, is truncated in the mitoribosome. This specific bacterial RNA–protein bridge is replaced by a protein–protein bridge in the mitoribosome. Despite the significant loss of RNA, the mammalian mitoribosome is bigger relative to the bacterial ribosome. Cryo-EM mapping of the mammalian mitoribosome revealed that the mRNA entrance site supports the recognition and translation initiation of mt-mRNAs that lack a 5′ untranslated region. Moreover, the P site in the large subunit structurally evolved to accommodate the mammalian mt-tRNAs, which are characterized by unusually small T loops [80]. It has also been proposed that the ribosomal exit tunnel on the large subunit evolved to facilitate the membrane binding in neosynthesized polypeptides that have a mitochondrial membrane function. This specific feature has been associated with the incorporation of mL45 protein to the perimeter of the exit tunnel and provides an evolutionary advantage since mitoribosomes synthesize mostly transmembrane proteins [81,82]. Notably, the recruitment of mL45 is an early event, which suggests that membrane binding in neosynthesized polypeptides was already apparent in the early stages of mitoribosomal evolution.

It is well accepted that the stability of ribosomes is mainly determined by rRNA secondary structures, followed by protein–rRNA interactions and by protein–protein interactions. In mammals, the mitochondrial genome encodes the 12S and 16S rRNAs that are involved in the assembly of the mt-SSU and mt-LSU, respectively, while all genes encoding the mitochondrial ribosomal proteins (MRPs) have been identified as nuclear. 5S ribosomal RNA (5S rRNA) is a universal structural component of bacterial, archaeal, and eukaryotic cytosol ribosomes and has a major role in the assembly of the central protuberance (CP) of the large subunit (LSU) [83]. However, in animals and fungi, no evidence has been found of an mtDNA-encoded 5S rRNA. Moreover, structural studies of mitoribosomes from yeast and mammals demonstrated the absence of 5S rRNA from the large subunit. Moreover, two proteins that make direct contact with the 5S rRNA in the eubacterial ribosome (L5 and L25) are also absent from mammalian mitoribosomes. It has been proposed that the altered MRP composition can compensate for the loss of this rRNA molecule. In agreement, the cryo-EM map of the mammalian mitochondrial ribosome has revealed that the stabilizing interactions normally provided by 5S rRNA have largely been replaced by MRPs [77]. In yeast, the structure of the large subunit suggests that the extension of specific mt-rRNA secondary structures may also contribute to this compensation [81].

The number of genes encoding MRPs in animals and fungi is significantly higher compared to in prokaryotes (90 genes have been identified in yeast, while only 54 have been identified in *Escherichia coli*) [84,85], and many of them are distinctive and lacking homology to the standard prokaryotic or eukaryotic set of MRPs [86]. This expansion on the number of mammalian MRPs initially led to the hypothesis that the higher protein content compensates for the loss of many RNA structural elements. However, the cryo-EM structure of the 55S mitoribosome revealed that many MRPs occupy new peripheral positions and that many rRNA reductions remain structurally unreplaced; for this reason, the core of the 55S mitoribosome is highly porous compared to the bacterial ribosome [87]. The integrated analysis of MRP and rRNA data from nuclear and mitochondrial genomes covering the eukaryotic domain indicates that the gain of MRPs occurred very early on in eukaryote evolution, before the divergence of the major eukaryotic lineages and before the evolutionary phase of the rRNA reduction that mainly characterizes the bilaterian metazoans [76,86].

Moreover, the mammalian MRPs that do not have clear eubacterial orthologues are evolving more rapidly than the proteins of the eukaryotic cytoplasmic ribosome [88]. The mammalian MRPs are imported into mitochondria and assemble with mitochondrially transcribed rRNAs into mitoribosomes. Therefore, the formation of mammalian mitoribosomes does not require the coexpression of rRNAs and MRPs in the mitochondrion. It has been proposed that shorter mitochondrial rRNAs have the potential to initiate ribosomal assembly without the presence of MRPs [75]. Mitochondrial gene expression and translation are central to cellular homeostasis and ATP production through oxidative phosphorylation. These mitochondrial-encoded polypeptides assemble with imported nuclear-encoded proteins and form multi-subunit protein complexes in the inner membrane of the organelle, which are involved in the OXPHOS machinery for ATP production [16]. The expression of these polypeptides from the mitochondrial genome is vital for the assembly and functioning of the OXPHOS complexes. Moreover, the dual origin of these complexes (nuclear and mitochondrial) poses a challenge for the cell, as the cellular production of energy requires the coordination of the cytosolic and mitochondrial translational programs for a balanced assembly of OXPHOS subunits (see Section 4).

## 4. Mitonuclear Coevolution

The formation of protein import systems was a major step in mitochondrial evolution because it permitted the appropriate transport of nuclear-encoded proteins in parallel with the massive mitochondrial gene transfer to the nucleus. This system consists of translocase complexes of the outer membrane (TOM) and translocase complexes of the inner membrane (TIM) that recognize a variety of targeting or sorting signals in mitochondrial proteins that are conserved in eukaryotes. Importantly, there is extensive crosstalk and exchange between the TOM and TIM import pathways.

A major class of targeting signals includes cleavable amino-terminal pre-sequences of 15–50 amino acid residues that form positively charged amphipathic α-helices. Upon import into the matrix, these pre-sequences are proteolytically removed by the dimeric mitochondrial processing peptidase (MPP). However, a large number of inner membrane mitochondrial proteins contain a pre-sequence-like signal that is often preceded by a hydrophobic sequence that is located within regions of the mature protein that are non-cleavable [89,90,91,92]. Tom40, an integral membrane protein with a β-barrel structure, is considered as the central component of TOM, forming the channel for preprotein translocation across the outer membrane. Two other receptor proteins, namely, Tom20 and Tom22, mediate the initial recognition of pre-sequences, which is necessary before directing the imported proteins to the Tom40 channel. 

TIM23, which is a pre-sequence translocase-associated motor (PAM) complex, and the TIM22 complex are the major translocase complexes of the inner membrane. The TIM23–PAM complex directs the pre-sequence-containing precursor proteins to the inner membrane or matrix, while the TIM22 complex imports hydrophobic membrane proteins, particularly mitochondrial carrier proteins. Even though translocase complexes recognize the conserved import signals, the display sequence variation, and diversity among eukaryotic lineages, only two subunits, namely, Tom40 and Tom22, are conserved [93]. Mitochondria encode a tiny subset of the proteins they need to function (~1%) [94]. As a result, there are at least three major mitochondrial processes that require nuclear- and/or mitochondrial-encoded proteins to participate: OXPHOS, mitochondrial translation, and mtDNA repair processes (the latter is discussed in the next section). This necessitates a level of co-operation and regulatory communication between these two cellular compartments to safeguard all the components for each process such that they are quantitatively, qualitatively, and promptly available. Any given mutation that could pose a challenge to this mitonuclear cooperation would compromise the faithful execution of each process. Given that mtDNA is mutated at a >20-fold higher rate than nuclear DNA, the two genomes must somehow manage to co-evolve to preserve this mitonuclear cooperation. 

Even though more evidence is required, several current lines of evidence underscore the tight coordination of the two evolving genomes in a range of aspects. For instance, numerous protein–protein interactions between mitochondrial- and nuclear-encoded proteins involved in the OXPHOS machinery were found to co-evolve [95,96,97,98,99,100]. In addition, mitochondrial rRNAs seem to co-evolve with nuclear-encoded ribosomal proteins to ensure structural and functional ribosomal integrity, where it has been observed that nuclear-encoded cytosolic ribosomal proteins are mutated at significantly lower rates compared to the mitochondrial ribosomal ones [101].

On top of the structural–functional preservation between proteins of the two compartments, their co-evolution needs to be integrated at the regulatory level as well. Indeed, although mtDNA-encoded mRNAs are polycistronic and translated within the mitochondrion, nuclear-encoded mitochondrial genes are scattered throughout chromosomes such that the spatial co-expression of all genes encoding subunits of a certain OXPHOS complex has been observed [102,103]. More recently, it was shown that the mitochondrial protein synthesis machinery is synchronized with the nuclear-encoded OXPHOS subunits, possibly through signals during the import of the latter into mitochondria [104,105]. Similar synchronization has been observed in yeast [106]. Seen from another angle, in type 2 diabetes patients, the deregulated expression of genes related to OXPHOS is also tightly orchestrated [107], suggesting that all these genes are co-regulated, and thus, co-deviate. Such coordinated responses are possibly linked with mitochondria-to-nucleus (retrograde) communication signals [108,109,110] or microRNA regulation [111]. An additional example of retrograde communication was recently provided by Huang et al., where mitochondrial ribonuclease T2 was shown to selectively degrade cytosolic ribosomes of the outer mitochondrial membrane and that this also triggered new rRNA transcription in the nucleus [112]. Another possibility for the coordinated regulation of gene expression in these two compartments is the utilization of the same transcription factors for both genomes. Prime candidates are the TFAM, POLRMT, c-Jun, Jun-D, and the thyroid hormone and glucocortinoid receptors [113,114,115,116,117,118].

Interestingly, further evidence for the mitonuclear co-evolution can be deduced from observations within and between species. For instance, one of the abovementioned transcription factors, POLRMT cannot stimulate the transcription of mtDNA-encoded genes of another species (at least between humans and mice) [119]. Even primate mtDNA cannot compensate for the loss of human mtDNA in human cell lines. The closer the species is to humans (chimpanzee, gorilla), the less severe the loss of OXPHOS function (of note, in orangutan, the most divergent primate from humans, mtDNA was unable to be maintained in human cells) [120]. On the other hand, intra-species variations have also been observed and involve the divergent degree of predisposition to several human pathologies of a given mitochondrial mutation by distinct populations (described in detail in Section 6.1). Similarly, a very recent report highlights that mutant mtDNA cannot adapt in non-permissive nuclear DNA in mice, i.e., if not co-evolved, nuclear and mitochondrial genomes are not able to support efficient mitochondrial function [121]. Similar incompatibilities between the two genomes have been observed in several other species (reviewed in [122]).

## 5. Mitonuclear Communication 

### 5.1. Mitonuclear Communication in Homeostasis and Stress

Since mitochondria participate in crucial cellular processes, such as energy production and intermediate metabolism, nucleus-to-mitochondria (anterograde) and mitochondria-to-nucleus (retrograde) communication safeguards cellular fitness. Anterograde regulation can decrease or increase mitochondrial activity, as well as promote mitochondrial biogenesis, depending on cellular needs. Conversely, mitochondria can coordinate retrograde signaling to the nucleus to alter nuclear gene expression and cell metabolism.

In the context of anterograde regulation, the transcription of nuclear-encoded mitochondrial proteins is mainly mediated by the nuclear transcription factors NRF1 and NRF2α. Both factors control the expression of cytochrome c and other nuclear-encoded OXPHOS subunits, as well as the expression of proteins implicated in the import system and mtDNA replication, transcription, and translation, such as mitochondrial ribosomal proteins and tRNA synthetases [123,124]. The transcription of nuclear-encoded mitochondrial proteins is also regulated by nuclear receptors, such as ERRα, -β, and -γ, as well as PPARδ. These factors promote the expression of proteins that are involved in the tricarboxylic acid (TCA) cycle, OXPHOS, and fatty acid oxidation [125,126]. Within the context of transcriptional regulation by these factors, PPARγ coactivator 1α (PGC1α), PGC1β, and the PGC-related co-activator PRC are known to positively regulate the function of NRF, ERR, and PPAR factors to induce mitochondrial biogenesis [127]. Conversely, anterograde signaling driven by TP53 can also lead to the transcriptional suppression of PGC1a and PGC1β and the reduction of mitochondrial metabolism in the case of high levels of DNA damage and nuclear stress [128].

Retrograde signaling is present under mitochondrial stress. OXPHOS dysfunction or high levels of mitogenome damage can trigger the loss of mitochondrial membrane potential and the release of Ca^2+^ into the cytoplasm [129,130]. Subsequently, elevated free cytosolic Ca^2+^ levels activate the phosphatase calcineurin and other Ca^2+^-regulated kinases, which in turn, can activate a wide panel of transcription factors, such as the nuclear factor-κB (NF-κB), the cAMP-responsive element-binding protein CREB, the early growth response protein 1 (EGR1), the cAMP-dependent transcription factor ATF2, the CCAAT/enhancer-binding protein-δ (CEBPδ), and the CEBP homologous protein (CHOP). The type of transcription factors that become activated depend mostly on the cell type, and the associated transcriptional changes can affect insulin signaling, glucose metabolism, and cell proliferation [131,132]. 

Another distinct pathway for retrograde communication is the mitochondrial ROS signaling. For instance, in *Caenorhabditis elegans,* high ROS levels under mitochondrial stress conditions can stabilize and activate the transcription factor hypoxia-inducible factor 1 (HIF-1), which regulates the adaptation to low oxygen levels. This signaling axis has also been associated with longevity [133,134]. Experiments in *Drosophila melanogaster* have demonstrated that increased ROS trigger cell cycle arrest through TP53 or JNK pathway activation [135,136]. In mammals, retrograde signaling through ROS has been correlated with the transcriptional upregulation of antioxidant proteins [137,138], while in cancer cells, it has been correlated with NF-κB activation and cellular proliferation and survival [139].

The mitochondrial electron transport chain (ETC) leaks electrons and generates reactive oxygen species (ROS) during normal respiration. When ETC function is deregulated, electron transfer slows and respiratory complexes become more highly reduced, which increases their reactivity with oxygen. As a result, an elevation of ROS production occurs. The mitochondrial theory of aging states that physiological decline with age is a result of the cumulative effects of damage from oxygen radical production [140]. In fact, it has been suggested that ROS could be responsible for aging by acting as a mutagen on mt-DNA. Mutations in a mitogenome lead to defective ETC, which in turn, produces even more damaging ROS. An excess of free radicals oxidizes membrane lipids, releasing cytochrome c, which is an event that initiates programmed cell death (apoptosis) in most eukaryotic cells [141]. Consequently, apoptosis can lead to aging-related tissue loss and inflammation in metabolically active tissues, such as brain and muscle. 

The mutagenic effect of ROS in mitogenome can also affect the fidelity of mitonuclear communication in somatic cells. Since ROS-driven mutations of mt-DNA cause age-related heteroroplasmy, a mitonuclear mismatch in somatic cells can also deregulate ETC function, which increases ROS leakage and the apoptotic rate [142].

While mitochondria are the primary source of ROS, the redox balance is regulated by a network of nuclear-encoded antioxidant proteins, such as mitochondrial Mn superoxide dismutase (SOD), cytoplasmic Cu/Zn superoxide dismutase, and glutathione peroxidase, which suppress ROS damage [143]. Thus, the proposed free radical model of aging states that longevity can be extended either via increased activity of the antioxidant pathways or via decreased levels of ROS production. Experimental studies suggest that the balance between ROS production and antioxidant activity affects longevity differently between species. For instance, calorically restricted mice live longer and produce lower levels of ROS, while caloric restriction does not decrease ROS production in *Drosophila melanogaster* [144]. Moreover, the effects of oxidative stress in aging may vary between individuals, as well as between sexes. Under oxidative stress, female *Drosophila melanogaster* express higher levels of antioxidant enzymes and display longer lifespans compared to males [145]. Similarly, female Wistar rats exhibit higher gene expression levels of antioxidants, lower oxidative damage, and longer lifespans than males [146]. Sex-biased gene expression under oxidative stress was also identified in copepod *Tigriopus californicus*. More specifically, females were found to display a more targeted response to oxidative stress by differentially expressing fewer genes but with a greater magnitude of fold change [147].

In humans, alternative mtDNA haplotypes are associated with variations in longevity [148,149,150]. Similarly, in *Drosophila melanogaster*, mitochondrial haplotypes have been found to affect the mtDNA copy number and mitochondrial gene expression, and are associated with sex-specific effects on fertility and longevity [151]. In this context, further studies in *Drosophila* revealed that mtDNA haplotypes affect aging in a nuclear-background-dependent manner [95,152] and this kind of mitonuclear epistasis could explain why some mtDNA mutations can have very different phenotypic effects in different individuals [153].

### 5.2. Mitonuclear Communication in mtDNA Damage and Repair

The abovementioned increased mutation rates on the mitochondrial genome compared to the nuclear ones were initially considered the outcome of its proximity to the electron transfer chain (i.e., due to an increased rate of oxidative damage) and the presence of poor or limited DNA repair systems in mitochondria. Proximal reactive oxygen species, which are byproducts of the mitochondrial respiration chain, such as superoxide (O^−^) and, in the presence of ferric iron or superoxide dismutase (SOD), its derivatives hydroxyl radicals (HO) or hydrogen peroxide (H_2_O_2_), respectively, can react with DNA bases (especially guanine and thymine to produce 8-oxo-7,8-dihydroxyguanine (8-oxo-dG) and 5,6-dihydroxy-5,6-dihydrothymine, respectively). However, O^−^ is not an efficient DNA modifier [154,155], and H_2_O_2_ diffuses immediately out of mitochondria [156]. Moreover, only ~0.1% of cellular oxygen was estimated to be converted to O^−^ [157], and once formed, it is rapidly converted to H_2_O_2_, either in the mitochondrial matrix (by SOD2) or in the intermembrane space (by SOD1) [158]. Of note, mtDNA is packed and protected by transcription factor A of mitochondria (TFAM) in nucleoids that, despite their proximity with the respiration chain, limit ROS’ access to mtDNA [159,160]. An elegant recent in vivo study by Kauppila et al. demonstrated that mitochondrial point mutational rates are indistinguishable between wild-type and single or double OGG1, MYH, and SOD2 knockout mice [161]; this aids the utilization of Polγ as the major acting polymerase in mitochondria. Polγ shares one of the higher efficiencies among DNA polymerases. In the cases of 8-oxo-dG lesions, which are generally considered to be highly mutagenic, this leads to G→T transversion mutations [162]. Polγ was found not to incorporate nucleotides opposite 8-oxo-dG, and when that was done, in most of the cases, the correct cytosine was incorporated instead of adenosine, which explains the low G→T frequency in mitochondria [163,164]. This notion is supported by the work of Kennedy et al., where the duplex sequencing technique was utilized, which suggests that the major source of point mutations in mitochondria is spontaneous deamination instead of oxidative damage [165]. Their data could also explain why the H-strand is heavier than the L-strand (the common G→A and T→C transitions occurring in the L-strand gradually lead to guanine enrichment of the H-strand). Moreover, alkylation (e.g., O6-methylguanine) and other bulky DNA adducts are also included in the list of damaging lesions observed in mtDNA. 

On the other hand, the repair pathway options responsible for reverting the increased DNA lesion load in mitochondria are not as limited as once thought [166,167]. The fact that mtDNA repair relies on proteins of nuclear origin remains unaltered, but in the last decade, increasingly more nuclear repair proteins with a diverse range of repair pathways have been identified in mitochondria. Base excision repair (BER) remains the most prominent and well-studied repair option acting on mitochondrial genome; however, additional pathways have been identified to supplement the mitochondrial repair arsenal (reviewed extensively in [168]). Mitochondrial BER is conducted similarly to the nuclear one, although not all nuclear proteins have been identified in mitochondria. For example, out of the 12 known mammalian glycosylases responsible for damaged base recognition and cleavage, seven act on mitochondria as well [169]. The translocation of repair proteins to the mitochondria depends on the presence of the mitochondrial targeting signal (MTS) or the MIA pathway [170,171]. Interestingly, some nuclear and mitochondrial repair proteins are differentially expressed; some are alternatively spliced (one isoform is active in the nucleus and the other in the mitochondria), e.g., the glucosylases uracil-DNA glycosylase (UDG), 8-oxoG DNA glycosylase 1 (OGG1), and MYH DNA glycosylase (MYH) or the endonuclease apurinic/apyrimidinic endodeoxyribonuclease 1 (AP1) [172,173,174], while some mitochondrial isoforms still contain the nuclear localization signal as well (e.g., OGG1 or NTH1) [175,176]. Others are the same in both compartments, to the degree they have been investigated, and few have a unique function on the mitochondria (e.g., the endonuclease exo/endonuclease G (EXOG1) [177]. BER is utilized to repair a damaged base (oxidized, alkylated, hydrolysed, deaminated, etc.). Besides BER, mitochondria employ direct reversal (DR), translation synthesis (TS), double-strand breaking and possibly mismatching (MMR), and nucleotide excision (NER) repair pathways to repair a range of lesions (Table 1) (reviewed extensively in [168,178]).

**Figure 4:**
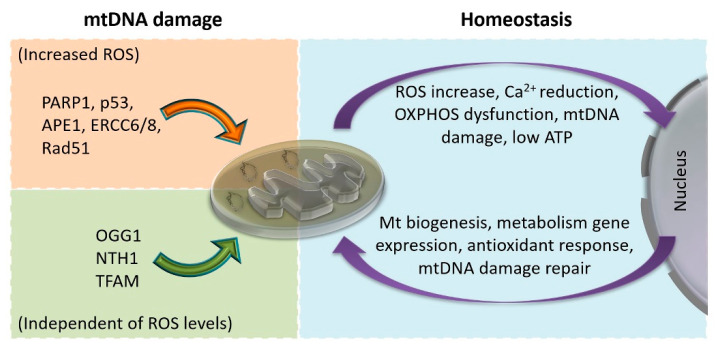
Cellular response to mitochondrial dysfunction. Left: Key molecules that translocate into mitochondria upon mtDNA damage in the presence or absence of increased reactive oxygen species (ROS) levels. Right: Sensed mitochondrial deviations and responses during homeostasis. OXPHOS: Oxidative phosphorylation.

Despite all the efforts toward the identification and characterization of repair proteins/pathways that participate in mitochondria, the most intriguing and obscure point in mtDNA repair is the DNA damage-sensing/response process. It remains unclear how the mtDNA lesion sensing is transferred to the nucleus or cytoplasm to initiate the suitable repair response, i.e., expression of the appropriate repair proteins or translocation of readily available proteins from the cytoplasm into mitochondria. It is evident that some BER proteins have a dual function in the nucleus and mitochondria, such as APE1 [179], Cockayne syndrome A and B type proteins (ERCC6/8) [180,181], and Rad51 [182], which are continuously present in the cytosol but can also translocate into mitochondria under an oxidative stress condition. Others, such as OGG1 and NTH1, respond only to the presence of mtDNA damage irrespective of the ROS levels for their translocation to the mitochondria [183,184,185]. Poly(ADP-ribose) polymerase (PARP1), which is a prominent regulator of nuclear repair, is also one of the first proteins that translocate to the mitochondria upon oxidative stress. However, it is unclear whether nuclear and mitochondrial PARP1 are the same or are distinct isoforms, while its action in mitochondria, although not clearly elucidated, seems strikingly different than in the nucleus since it was shown to negatively regulate mtDNA repair [186,187,188]. PARP1 interacts with several BER or NER repair-related proteins in mitochondria (Polγ, EXOG1, TFAM, CSB, APTX, and TDP1), as well as numerous other proteins or enzymes of the respiratory chain or Krebs cycle, leading to reduced replication/transcription/respiration activities and NAD+ and acetyl-CoA exhaustion. On the other hand, NAD+ and acetyl-CoA exhaustion trigger a cascade of events, initially through sirtuins, that leads to the expression of numerous repair and oxidative stress response proteins in the nucleus (reviewed extensively in [189]). Another known protein that is transported to the mitochondria upon oxidative stress is p53 [190]. p53 is actively involved in several modes of mitochondrial BER through its interactions with the single-stranded DNA binding protein (SSB), Polγ, OGG1, APE1, and TFAM [191]. The latter is also suggested as an initial mtDNA damage response regulator, which, upon taking damage, seems to restrict replication/transcription/repair processes to provide the necessary window for the translocation of repair factors into mitochondria and to assess the extent of DNA damage [192]. The major mtDNA damage response molecules that are identified in damaged mitochondria, as well as the cellular response to sensing mitochondrial dysfunctions, are depicted in Figure 4.

All the above repair processes can also be seen as the result of the continuous co-evolution of mitochondrial and nuclear genomes toward the beneficial goal of preserving “pan-cellular” genomic integrity, and as a result, cellular fitness. Previously presented evidence, such as the identification of mitochondrial-specific isoforms of repair enzymes or the unique mitochondrial action of EXOG1 and the evolution of MTS or other import mechanisms, can only be taken as a co-evolution proof of concept. Additional evidence can be deduced from ablation experiments on several key repair components, such as PARP1 [186], p53 [193,194], APTX [195,196], or TDP1 [197], where their role in the relevant repair process cannot be compensated for, which is indicative of their unique but coordinated evolution and specific function in either compartment. 

Collectively, the source of DNA damage in the mitochondrial genome cannot be solely attributed to their intense respiratory activity or poor/limited repair. The increase in oxidative damage with age is well documented [198], but evidence of increased mitochondrial ROS production acting as an aging driver is missing. Additionally, since they utilize Polγ, which is one of the best polymerases in terms of processing fidelity, the increased mutational frequency due to replication errors should also be excluded. Based on the observations of [165] and the asymmetric-strand-specific mutational load in human cancers [199,200], the strand-specific mode of mtDNA replication, as previously suggested [168,201,202,203], could be a prime candidate for the observed increased mutational load of the mitochondrial genome in either cancerous or aged human cells.

## 6. Defects in Mitochondria in Aging and Disease

**Figure 5:**
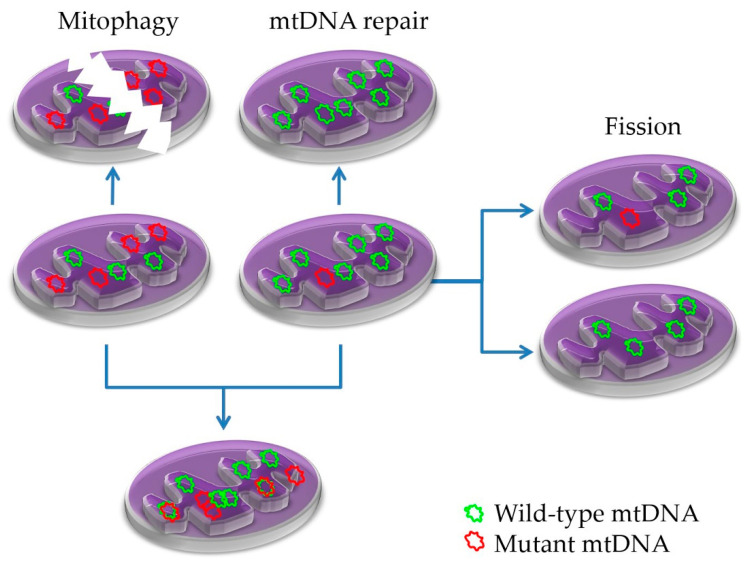
Mechanisms of mitochondrial quality maintenance. Mitochondria contain several copies of mtDNA. Mutant mtDNA copies replicate and heteroplasmic mitochondria emerge unless repaired. Through fusion and fission events, heteroplasmic levels increase or decrease. Increased mutant mtDNA bearing mitochondria can be eliminated through mitophagy.

Mitochondrial integrity is maintained by a range of processes involving mitochondrial DNA damage repair, fusion, and fission, with mitophagy (autophagy of mitochondria) being prominent (Figure 5) [204,205,206,207,208]. Deregulation of any of the above quality control processes may cause mitochondrial impairments, which lead to or contribute to several pathologies, including neurodegenerative and muscular disorders, cancer, and aging [209,210,211].

Mutations in mtDNA or nuclear-expressed mitochondrial genes impair mitochondrial function. Any given major mutation that leads to severe mitochondrial malfunction is negatively selected, underscoring the vital importance of this organelle to the cells. However, due to heteroplasmy, divergent ratios of mtDNA mutations or polymorphisms are present in any given individual or even among cells of a given organ. The source of this variation originates from the degree of heteroplasmy that the zygote will inherit from its mother (oocyte) and the selection process (genetic bottleneck) that followed during the primordial germ cell development (reviewed in [212]). In brief, during female germline development, a huge reduction (1000-fold) in the mtDNA copy number per cell occurs (bottleneck), which is followed by a sharply divergent heteroplasmy level among daughter cells. This is considered a genetic drift-driven process, though it is difficult to be thoroughly investigated in animals. A recent work by Wei et al. investigated the maternally transmitted heteroplasmy and not only found that the human population mitogenome is genetically selected during heteroplasmy transmission in female germ cell development but also that the selected mtDNAs are influenced by the nuclear genetic background [213]. Seeing these findings from another angle, this bias imposed by the nuclear genetic background is another “proof of concept” for the co-evolution of the two genomes. 

From an evolutionary endpoint, the ancestral human mtDNA originates from a female of African origin ~200,000 years ago. These ancestors evolved regionally for more than 150,000 years, generating an array of distinct haplogroups (L0, L1, L2, etc.). Only two of the L3 mtDNA haplogroups colonized the rest of the world, Europe and Asia (N macrohaplogroup) and Southeast Asia (M macropaplogroup) initially, and from Asia (haplogroup derivatives of the N and M macrohaplogroups) to America later on (about 25,000 years ago) [214]. Such evolution/adaptation events led to the generation of unique regional haplogroups with a distinct predisposition to several human pathologies. For example, individuals of the European haplogroup H5a bearing the *MT-TQ* m.4336A>G mutation, or the UK haplogroup are predisposed to late-onset Alzheimer’s disease [215,216,217], while H haplogroup individuals are predisposed to Parkinson’s disease [218]. Other haplogroups, such as T and J, are associated with a decreased risk of either Alzheimer’s or Parkinson’s diseases [219,220]. Similar evidence has been provided for an array of additional neurodegenerative diseases, such as autism, and metabolic diseases, such as diabetes, hypertension, and obesity (reviewed in [214]). 

Apart from such widely fixed mtDNA mutations, point mutations may develop spontaneously and colonize a cell, an organ, or even an entire individual if inherited or formed de novo in the oocyte or early embryo. If they do not interfere with (suppress) mtDNA replication, unrestrained heteroplasmies will emerge following neutral drift [221]. Low-level heteroplasmies can be found in almost every healthy individual [222], whereas some of them could also be pathogenic [223]. However, even if a mutation targets an important mitochondrial function (e.g., OXPHOS), to be effective, it needs to increase its presence through successful replication cycles beyond a certain threshold. For example, most pathogenic OXPHOS mutations are ineffective in the range of 0–60% heteroplasmy [224]. 

### 6.1. Human Mitochondrial Diseases

As mentioned earlier, founder mutations affect certain populations differently and may also provide different predispositions to other pathologies. These phenomena became evident in the past decade with the aid of advanced next-generation sequencing technologies and underline the environmental contribution in the evolution of mtDNA. It has been estimated that mitochondrial diseases affect about 11 out of 100,000 adult individuals, but the ratio becomes much higher in population-based studies, as in the case of the mitochondrial encephalomyopathy, lactic acidosis, and stroke-like episodes syndrome (MELAS) *MT-TL1* m.3243A>G mutation, which reaches ≈236 cases per 100,000 individuals in an Australian-Caucasian cohort (Table 2) [225]. However, recent evidence revealed that 1 in 200 newborns bear a pathogenic mtDNA mutation [226] and that each individual bears on average one heteroplasmic mutation, whereas one in eight individuals harbors a mitochondrial disease heteroplasmic mutation at >1% heteroplasmy [227]. It is also important to stress that mtDNA mutations are responsible for 80% of adult and only 20–25% of childhood mitochondrial diseases (see below), rendering autosomal recessive nuclear DNA mutations responsible for the vast majority of mitochondrial diseases in children [228]. Moreover, most of the mitochondrial human diseases are linked with mutations in respiratory or tRNA-related genes than in rRNAs or mitoribosomal proteins [229].

In general, mitochondrial diseases can be categorized into two groups, those with onset during the first 3 years of life (childhood) and those with onset at about 20–40 years (adulthood). Most mitochondrial diseases affect multiple organs (rarely only one) and are difficult to be treated since a given genotype is often associated with a range of clinical phenotypes. For example, the *MT-TL1* m.3243A>G mutation is present in patients with chronic progressive external ophthalmoplegia (CPEO), MELAS syndrome, and maternally inherited diabetes and deafness (MIDD) [230]. The early onset mitochondrial diseases include the Leigh, Alpers–Huttenlocher, and Pearson syndromes, with the former being the most common (Table 2) [231]. In general, they are severe diseases (often fatal) and often co-exist with other clinical pathologies (e.g., of the heart). Leigh syndrome, the most common mitochondrial-related childhood disease, is linked with mutations in at least 75 genes and mostly affects the brain [232]. Interestingly, although the rate of Leigh syndrome is ≈2.5 cases per 100,000 births, in a specific Canadian population, it was 10-fold higher due to a founder mutation in *LRPPRC*. Mutations in the *POLG* gene or the tRNA synthases *NARS2* and *PARS2* are found with Alpers–Huttenlocher syndrome [233,234], while other, less common, mitochondrial (childhood) syndromes are related with mutations in *TK2*, *SERAC1*, or *AGK*, and the depletion of mtDNA [235,236,237]. 

Mitochondrial diseases of adults include Leber hereditary ocular neuropathy (LHON), MELAS, and Kearns–Sayre syndromes, as well as neurogenic muscle weakness, ataxia, and retinitis pigmentosa (NARP) and CPEO. LHON syndrome affects retinal nerve cells and causes blindness. Interestingly, different founder mutations across distinct populations are responsible for this syndrome, while sex and other habits, such as smoking or alcohol consumption, are strongly associated with the disease outcome [238,239,240]. Approximately 80% of MELAS syndrome patients bear the *MT-TL1* m.3243A>G mutation mentioned above. Their symptoms include myopathy, encephalopathy, and stroke-like episodes [241]. Kearns–Sayre syndrome, the first human disease that was linked to mitochondria, has a common genetic origin with Pearson syndrome and CPEO, extensive deletion and mtDNA rearrangement, and is characterized by retinitis pigmentosa, external ophthalmophegia, and complete heart blockages [242]. NARP is caused by *MT-ATP6* m.8993T>G or m.8993T>C mutations and often shows Leigh syndrome’s clinical features. Individuals with less than 70% heteroplasmy on m.8993T>G are usually asymptomatic, those with 70–90% suffer from NARP, while the ones with > 90% manifest clinically as Leigh syndrome [243]. On the other hand, carriers of the m.8993T>C mutation remain asymptomatic when bearing < 90% heteroplasmy. Finally, mutations in three-quarters of the known mitochondrial maintenance genes (tRNA genes included) have shown to cause CPEO, which is a condition that affects mostly muscles and vision [244,245].

### 6.2. Aging 

Aging is an inevitable process that affects almost all metazoans through their slowly progressive functional decline. In humans, this reduction is clearly evident in organs with high energy needs, such as the brain, muscles, and heart. Thus, it is not surprising that most mtDNA mutations were observed in aged tissues from the brain, retina, skeletal muscles, heart, hepatocytes, and gametes [246,247,248,249,250,251]. These mutations are usually large-scale (at the kilobase level) deletions, while debate continues regarding whether other mutations (point mutations) contribute to aging [252,253]. Although tissue homoplasmies are less than 10% for most of the observed deletions, specific defective tissue regions seem to have the highest levels [254,255,256]. The origin of these age-associated mtDNA deletions (inherited or acquired de novo) may be a matter of debate [257], but their strong presence in numerous aged organs is undisputed; their frequent presence in a range of age-related human pathologies, especially neurodegenerative ones, is also undisputed. Again, the causal role or contribution of them remains unresolved since, for example, mtDNA mutations observed in Parkinson’s disease are also present in healthy-aged human dopamine neurons, though at lower levels [258]. 

### 6.3. Cancer

Until recently, mtDNA mutations were thought to actively participate in cancer development and progression. This notion was supported by the fact that many cancers ferment glucose to lactate. Mitochondrial respiration defects were suggested by O. Warburg to be responsible for this unusual property back in the mid-1950s [259]. More recently, numerous reports on disputable mtDNA sequencing data support a significant role of mitochondria in human malignancies [260]. However, recent studies on TCGA cohorts of various human cancers revealed that deleterious, pathogenic mutations in the mitochondria of cancer cells were negatively selected and remain heteroplasmic, whereas missense mutations were reaching homoplasmy due to neutral drift selection [199,200,261]. Therefore, it is reasonable to assume that human cancers tend to retain healthy mitochondria that are free of harmful mtDNA mutations. 

To this end, further supporting evidence came from functional studies, where the depletion of mitochondria, through the inactivation of TFAM, restrained tumor growth and Kras-mediated tumorigenicity in lung tumor models [262]. Similarly, the generation of ρ0 lung cancer cells leads to the loss of their tumor-initiating potential [263]. Moreover, another set of compelling evidence over the minimal contribution of mitochondrial mutations in human malignancies came from studies in oncocytomas. Oncocytomas are benign tumors with a vast accumulation of mitochondria bearing pathogenic mtDNA mutations. Primary oncocytomas are highly glycolytic, suggesting that impaired respiration renders them unable to progress from a benign stage [264]. Additionally, they have highly defective Golgi and vesicle trafficking, implying abnormal intracellular signaling, including mitonuclear communication [264]. Additionally, experiments in lung mouse models revealed that the depletion of autophagy regresses the tumors onto benign oncocytomas and that preserving mitochondrial glutamine metabolism is a necessary step in Braf^V600E^-driven lung tumorigenesis [265,266].

On the other hand, increased ROS levels are evident in most human cancers. mtDNA mutations that affect respiration and lead to increased ROS levels seem to favor some malignant neoplasms, such as head and neck squamous cell carcinomas or lung carcinomas [267,268,269]. Similarly, ROS-promoting mutations are present in mitochondrial diseases, such as NARP, Leigh syndrome, and other neurological pathologies [270,271,272]. However, compelling in vivo evidence for mtDNA mutations and cancer was given in recent reports on prostate and renal cancer [273,274]. Both works support the notion that the observed mutations may indeed have a driver role in those malignancies. Additionally, as mentioned earlier, according to TCGA data, numerous mtDNA mutations are observed in all human solid tumors [199,200,261]; however, given the low heteroplasmy extent of the pathogenic mitochondria, the vital importance of functional mitochondria for a cell and the lack of solid experimental evidence for the role of mtDNA mutations in cancer makes it difficult to decipher the extent of their contribution. 

All the above data suggest that cancer cells, similar to healthy ones, depend on functional mitochondria for their maintenance and progression toward more aggressive phenotypes. Their unique tumor microenvironment provides additional selective pressure and mtDNA mutations that reach increased heteroplasmy ratios or even homoplasmy to ensure adequate mitochondrial functional fidelity. As explained above, cancer cells bypassing these restrictions, in vivo or in experimental settings, lose their tumorigenic potential and regress to a benign state where they can not progress further from it in most cases.

## 7. Concluding Remarks

Mitochondrial translation has evolved to be coordinated by mitonuclear interactions to respond to the energetic demands of the cell. These interactions provide a level of cooperation and regulatory communication between these two cellular compartments to safeguard all the components for each process such that they are quantitatively, qualitatively, and promptly available. Mutations in mtDNA or nuclear-expressed mitochondrial genes impair mitochondrial function. The loss of mitochondrial integrity is evident in aging, as well as in numerous human pathologies.

## Figures and Tables

**Table 1:** Major mitochondrial repair pathways and key enzymes that act to revert the relevant DNA lesions.

Repair Pathway	Type of Damage	Key Enzymes
BER	Deamination	UNG
	Oxidation	MYH, OGG1, NTHL1, NEIL1, and NEIL2
	Alkylation	MPG
SSBR	Single-strand breaks	Utilizes BER components
MMR	Mismatch	MSH2, YB-1, NEIL2 and DNA ligase 3
DSBR	Double-strand breaks	SSB, MGME1, Rad51, Twinkle, and TFAM
NER	Bulky adducts	CSA, CSB

**Table 2:** Mitochondrial diseases, major involved mutations, and the estimated frequency in adults [225].

Syndrome	Mitochondrial Mutations or Mutated Nuclear Genes	Number of Cases per 100,000 Individuals (Adults)
**Leigh syndrome**	>75 genes	3.7
**Alpers**–**Huttenlocher syndrome**	*POLγ*	0.3
**Pearson syndrome**	Large mtDNA deletion or rearrangements	1.5
**LHON syndrome**	*MT-ND4* m.11778G>A, *MT-ND6* m.14484T>C and *MT-ND1* m.3460G>A	
**MELAS**	*MT-TL1* m.3243A>G	3.5
**Kearns**–**Sayre syndrome**	Large mtDNA deletion	1.5
**NARP**	*MT-ATP6* m.8993T>G or m.8993T>C	
**CPEO**	*TYMP, POLG1, POLG2, MPV17, SLC25A4, TK2, DGUOK, OPA1, AFG3L2, DNM2, RNASEH1, DNA2* or *C20orf7*	0.7

CPEO: Chronic progressive external ophthalmoplegia, LHON: Leber hereditary ocular neuropathy, MELAS: Mitochondrial encephalomyopathy, lactic acidosis, and stroke-like episodes, NARP: Neurogenic muscle weakness, ataxia and retinitis pigmentosa.
